# Supplementary material for: Inflammation and hemostasis in loiasis before and after ivermectin treatment: A biological pilot cross-sectional study
Source: PLoS Negl Trop Dis. 2024 Dec 16;18(12):e0012491. doi: 10.1371/journal.pntd.0012491 (PMC11684681; doi:10.1371/journal.pntd.0012491)
Supplement: Attachment — Submitted filename: Review PLOS NTD HECOLO v2.docx [file pntd.0012491.s001.docx]

Reviewers’ comments

Methods

Reviewer #1: The article does not clearly state why ivermectin therapy was chosen. The introduction should clearly state the specific advantages of this therapy over others.
Authors should indicate whether each type of loiasis treatment is associated with hemostatic disorders, or whether this is specific for the ivermectin therapy only.

It is a bit confusing, why patients with the highest MFD count (20,000-40,000) did not receive the treatment.

**Response**: Ivermectin was chosen because it is widely used to combat onchocerciasis, with single doses distributed every year in meso- and hyperendemic communities. In addition, due to its potent microfilaricidal effect, ivermectin can induce serious adverse events (SAEs) such as life-threatening encephalopathies in patients with high *L. loa* microfilarial densities (MFDs) above 30,000 mf/mL. As mentioned in the introduction, “Evidence from primate models suggests an association between post-ivermectin life-threatening encephalopathy and hemostatic dysregulation, characterized by degenerating mfs in small vessels associated with fibrin deposition, endothelial damages, and hemorrhages [9]. Similarly, humans experiencing post-ivermectin encephalopathy often exhibit conjunctival and retinal hemorrhages [10].”

Therefore, we decided to investigate a potential hemostatic dysregulation associated with ivermectin use in loiasis. For safety reasons, we chose not to administer ivermectin in individuals harboring MFDs above 20,000 mf/mL due to the risk of SAEs.

Two other drugs can be used to treat loiasis patients: diethylcarbamazine (DEC) and albendazole. DEC can also induce *Loa*-related SAEs, because of its microfilaricidal effect, but it is used only for individual treatment (mainly in travel medicine consultations), with prolonged 3–4-week courses to obtain a macrofilaricidal effect. DEC is not available in most of the African countries where loiasis is endemic. Albendazole has no microfilaricidal effect but, again, a 3-week course is needed to obtain a progressive decrease in *L. loa* MFD and its use is restricted to individuals with very high MFDs. Unlike ivermectin, data supporting a potential hemostatic dysregulation with the use of DEC or albendazole are scarce. Such a phenomenon is unlikely with albendazole, given its mechanism of action. It might be possible with DEC, and further studies could be considered as part of travel medicine consultations in non-endemic countries where detailed hemostasis investigations are easier to conduct (see below).

We added in the introduction: “IVM mass drug administration in onchocerciasis endemic regions may cause SAEs such as life-threatening encephalopathies in patients with high *L. loa* MFD above 30,000 mf/mL, due to its potent microfilaricidal effect”.

Results

Reviewer #1: Elevation of d-dimers, observed in this work, is an indirect sign of the hemostasis dysregulation. In order to evaluate the patients’ hemostasis state, global hemostasis test, sensitive to both hypo- and hyper-coagulation, like thrombin generation test, or thrombodynamics, should be assessed.

**Response**: We thank the reviewer for the valuable suggestion. This work was designed as a pilot biological study, as data on *Loa*-related hemostasis dysregulation remained unexplored in humans. In light of the D-dimers elevation observed in this work, we may consider the implementation of global hemostasis tests such a thrombin generation test and thrombodynamics in an upcoming study.

However, hemostasis tests such as thrombin generation and thrombodynamics are greatly influenced by preanalytical conditions and require high-quality plasma samples, which can be challenging to maintain under field conditions in rural areas of Central Africa. Indeed, these tests ideally require immediate centrifugation and storage at −80°C, which is currently unfeasible in the field. Additionally, logistical and time constraints must be considered, as frozen samples need to be sent abroad since such tests were not available in Cameroon.

Reviewer #3: -The authors describe an elevation of neutrophils in the results section, however only granulocytes are depicted in table 1. Can the authors add the neutrophil counts here?

**Response**: We appreciate the reviewer's keen observation. White blood cells were assessed with the HumaCount 30^TS^, a 3-part differential hematology analyzer which provides granulocytes counts without distinguishing neutrophils, eosinophils or basophils. Contrary to eosinophils which were manually counted with the use of Wright-stained blood smears, neutrophils counts were not assessed in this study. We would like to apologize for any confusion and appreciate the opportunity to rectify this error. We made the following changes:

- Lines 25, 130, 154 : “neutrophils” were replaced by “granulocytes”
- The paragraph discussing neutrophils involvement (lines 159-163) was deleted.

Reviewer #3: -In the first paragraph of the results section, the authors describe that ‘there was a trend towards increased leucocytes, neutrophils and eosinophils’. However, for eosinophils this is not only a trend, but a highly significant elevation. The text should be adjusted to make this more clear.

**Response**: We changed line 131 as follows to make the significant eosinophils elevation clearer. “Median eosinophil counts were significantly higher in patients with a MFD exceeding 20,000 mf/mL compared to those with no mf (1,016/µL vs 111/µL, p<0.001).”

Reviewer #3: -Platelets are significantly lower in 20-4,999 mf/mL group. Can the authors discuss on these findings?

**Response**: Platelet counts among groups were indeed significantly different (p=0.044), with medians notably higher in patients with high microfilarial counts (241,000 /µL in group C and 254,500 /µl in group D) than in those with no mf or with less than 5,000 mf/mL (180,000/µL in groups A and B).

We added the following elements in the manuscript:

- Results, line 132: “Similarly, median platelet counts were also significantly higher in the same group (254,500/µL vs 180,000/µL, p=0.044).”
- Discussion, line 161: “The notable increase in platelets with increasing MFDs suggests their probable role in filarial infections. Prior research has established that platelets mediate the immunological killing of *L. loa* mfs (14,15), while data in lymphatic filariasis also demonstrated mfs’ ability to modulate platelet functions (16).”

Conclusions

Reviewer #3: The conclusion (inflammation activation and pronounced fibrinolysis, indicative of coagulation activation) is mainly based on elevation of neutrophils and eosinophils, as well as D-dimer levels. More specific markers need to be included to be able to conclude this.

**Response**: We appreciate the valuable remark. However, our conclusions are based on alterations of a broad range of inflammatory markers such as eosinophils, CRP, ESR, albumin, fibrinogen, alpha-1-globulins and D-dimers. The variations observed in our study strongly support the existence of a systemic inflammatory response. Moreover, D-dimers elevation suggests both enhanced fibrinolysis and coagulation activation.

As this work was designed as a pilot biological study in the absence of pre-existing data, we may consider in upcoming studies with adequate sample sizes the addition of more specific markers (such as TAT-complexes, von Willebrand factor levels, thrombin generation and thrombodynamics tests), allowing us to fully investigate the mechanisms underlying hemostasis impairment.

However, advanced hemostasis tests mentioned above are greatly influenced by preanalytical conditions and require high-quality plasma samples, which can be challenging to maintain under field conditions in rural areas of Central Africa. Indeed, these tests ideally require immediate centrifugation and storage at −80°C, which is currently unfeasible in the field. Additionally, logistical and time constraints must be considered, as frozen samples need to be sent abroad since such tests were not available in Cameroon.

Summary and General Comments

Reviewer #2: Lepage et al. report an observational study in patients with loasis, in whom markers of inflammation and coagulation were measured. They observed that eosinophils increased with MFD. In a subgroup that received ivermectin treatment, an increase of eosinophils was also associated with an increase in inflammatory parameters and D-dimer. This is an interesting study, whose results improves the understanding of loiasis.

Major comments:
1. I find it difficult to understand, what is the authors’ main interpretation of d-dimer changes. The abstract suggests that it is increased fibrinolysis.  “Ivermectin… appeared to induce… pronounced fibrinolysis” (line 29,30). The paragraph about d-dimer changes in the discussion (line 176 ff) also starts with an interpretation of increased d-dimer as an indicator of increased fibrinolysis, then suggests that it might be indicative of increased thrombus formation / hypercoagulability. This is confusing, as a “pronounced fibrinolysis” would imply a bleeding phenotype. D-dimer is a compound biomarker of increased coagulation activation AND fibrinolysis activation which happen simultaneously. It is not a fibrinolysis biomarker, and – in the absence of other findings – is not indicative of thrombotic processes.

**Response**: We appreciate the valuable remark. We agree that fibrin degradation products such as D-dimers suggest both coagulation activation and biological fibrinolysis, which indeed happen simultaneously.

Regarding the reviewer’s comment about clinical phenotypes, we would like to remind that the follow-up was only biological and did not include any clinical outcome. As a matter of fact, the existence of an unreported thrombotic or hemorrhagic process cannot be formally ruled out.

*Modifications*:

- Line 29-30, the term “pronounced” which may wrongly suggest a clinical bleeding phenotype was removed. “Ivermectin treatment appeared to induce inflammation, coagulation activation and fibrinolysis”.
- Line 177, we replace the sentence by : “The increase in D-dimers observed on D4 post-IVM suggests both enhanced fibrinolysis and coagulation activation which happen simultaneously”

2. Laboratory analyses should be described in more detail. Information regarding reagents and analyzers should be provided. The method of fibrinogen measurement should be provided (Clauss method?).

**Response**: Thanks for the suggestion. Fibrinogen levels were indeed measured by Clauss method. Measurement methods of laboratory parameters were added to the manuscript.

Lines 92-104 were modified as follows: “Venous blood samples were collected from each participant using EDTA, sodium citrate, and dry blood tubes. Complete blood counts (flow cytometry), erythrocyte sedimentation rate (ESR) after one-hour (Westergren method), C-reactive protein (CRP) levels assessed by nephelometry, prothrombin time (PT), activated thromboplastin time (aPTT) and fibrinogen levels (chronometry) were measured at the hematology laboratory of the Yaoundé University Teaching Hospital. Wright-stained blood smears were prepared, and the percentage of eosinophils was recorded after examining 200 leukocytes. Eosinophil counts were calculated by multiplying the percentage of eosinophils by the white blood cells count.

Serum protein electrophoresis, ferritin (chemiluminescence) and D-dimers (ELISA) were assessed at the Centre Pasteur in Yaoundé. Finally, prothrombin fragment F1+2 (ELISA) and plasminogen activity (amidolytic method followed by nephelometry) were evaluated in a subset of participants (10 with no microfilariae and 10 with 20–40,000 mf/mL) at the Cerba Laboratory in France.”

3. Statistical analysis. Were the data tested for normality? Data are presented as mean and SD, but non-parametric tests were used to compare groups. Generally, normally distributed data should be presented as mean and SD, otherwise it is usually presented as median and IQR. Normally distributed data should be compared using parametric tests.

**Response**: We thank the reviewer for having raised this important point. Indeed, the data are non-normally distributed, and thus all tables were modified with results presented as median and IQR.

4. Presentation of results. Reference ranges for parameters should be provided in the tables. Age and sex of the study participants in each of the four cohorts should be provided in Table1. In Table 2, MFD at baseline and at D4 after treatment should be provided. The terms neutrophils or granulocytes should be used consistently. Some figures, e.g. box plots, or scatter plots for possible correlations should be considered.

**Response**: Thank you for the valuable suggestion. Age and sex were added in Table 1, and MFD was added in Table 2. Parameters’ reference ranges were also added in footnotes of both tables. Finally, as mentioned in reviewer 3’s comment on neutrophils, we made changes lines 25, 130, 154 where “neutrophils” were replaced by “granulocytes”.

Minor comments:
5. Abstract. Use of the term “correlation” wrongly suggests Spearman or Pearson analysis, when the observation in fact is, that granulocytes increase with MFD.

**Response**: We thank the reviewer for raising this point. We removed the term “correlation” and modified the sentence as follows: “At baseline, eosinophils (p<0.001) and platelets (p=0.044) increased with microfilarial densities.”

6. The introduction should include background information regarding ivermectin.

**Response**: Thank you for the suggestion. We added in the introduction: “IVM mass drug administration in onchocerciasis endemic regions may cause SAEs such as life-threatening encephalopathies in patients with high *L. loa* MFD above 30,000 mf/mL, due to its potent microfilaricidal effect”.

7. Why did the group with the highest MFD not received ivermectin treatment?

**Response**: Ivermectin may cause serious adverse events (SAEs) such as life-threatening encephalopathies in patients with high *L. loa* microfilarial densities (MFDs) above 30,000 mf/mL. Therefore, for safety reasons, we chose not to administer ivermectin in individuals harboring MFDs above 20,000 mf/mL.

8. Line 144, it should not be stated that F1+2 increased, as the p-value was quite high, and the number of patients (n=7) was small.

**Response**: We thank the reviewer for his/her vigilance. Line 147 was modified as follows: “Finally, prothrombin fragment 1+2 did not significantly vary (252 versus 469 pmol/L, p=0.313).”

Reviewer #3: Tristan Lepage and colleagues have investigated the impact of loiasis and its treatment with ivermectin on hemostasis and inflammation. It concerns a pilot study with a limited number of participants, and differences were observed in some general inflammation and hemostasis parameters. For me, the analyzed parameters are too general and conclusions are drawn by elevation of some of these parameters.
-Most importantly, coagulation activation is now suggested based on elevated D-dimer levels. Better markers, for examples TAT-complexes, should be provided to be able to demonstrate coagulation activation.
-Eligible participants for this study, screened for the presence of L. loa mfs, were stratified into 4 groups based on their MFD. However, how is it then possible to have a group with 0 mf/mL?
-Measuring von Willebrand factor antigen levels would be of added value for this study to demonstrate a dysregulation of hemostasis.
-An elevation of neutrophils is observed, and the authors aim to link L. loa to hemostasis and thrombosis. A discussion on the possible involvement of neutrophils extracellular traps (NETs; related to their role in coagulation activation) would be in place.

**Response**: We appreciate the reviewer's insightful comment. This work was designed as a pilot biological study, as *Loa*-related hemostasis dysregulation remained unexplored in humans. In the absence of pre-existing data, we decided to start with assessing general parameters in the look for any hemostasis impairment. Our study supports post-IVM alterations of several inflammatory markers (eosinophils, CRP, ESR, albumin, fibrinogen and alpha-1-globulins) as well as a significant D-dimers increase, suggesting both enhanced fibrinolysis and coagulation activation.

These results pave the way for upcoming studies with adequate sample sizes, and we may consider the addition of TAT-complexes, von Willebrand factor levels as well as thrombin generation and thrombodynamics tests as suggested by reviewer 1. However, advanced hemostasis tests mentioned above are greatly influenced by preanalytical conditions and require high-quality plasma samples, which can be challenging to maintain under field conditions in rural areas of central Africa. Indeed, these tests ideally require immediate centrifugation and storage at −80°C, which is currently unfeasible in the field. Additionally, logistical and time constraints must be considered, as frozen samples need to be sent abroad since such tests were not available in Cameroon.

Regarding the study population, adults aged 25 to 70 were first screened for *L. loa* mfs. Based on the results of *L. loa* MFD, eligible participants were stratified into 4 groups, including a control group with negative MFDs (0 mf/mL). The term “the presence of” line 64 was removed to avoid any confusion.

Finally, we would like to apologize once again for the incoherence as only granulocytes –and not neutrophils– were assessed in this study. Mentions of neutrophils elevation were removed from the manuscript.
